# Differentials of modern contraceptive methods use by food security status among married women of reproductive age in Wolaita Zone, South Ethiopia

**DOI:** 10.1186/s13690-015-0089-5

**Published:** 2015-09-28

**Authors:** Mohammed Feyisso, Tefera Belachew, Amanuel Tesfay, Yohannes Addisu

**Affiliations:** Department of Public Health, College of Health Sciences and Medicine, Dilla University, Dilla, Ethiopia; Department of Population and Family Health, Jimma University, Jimma, Ethiopia

**Keywords:** Modern family planning, Currently married women, Food insecurity, Soddo Zuria woreda

## Abstract

**Background:**

In spite of the massive spending and extensive family-planning promotion, many poor people in the third world remain reluctant to use modern contraceptive method. Mostly when they use modern contraceptives, their continuation rates are often low. Reproductive health can improve women’s nutrition; in return better nutrition can improve reproductive health. Thus addressing the connection between nutrition and reproductive health is critical to ensure population growth that does not overwhelm world resources.

**Methods:**

A community based cross-sectional study was conducted from March 15–30, 2014 in Soddo Zuria Woreda, Southern Ethiopia. A total of 651 currently married women of reproductive age group were selected using multistage sampling. Probability proportional to the size allocation method was employed to determine the number of households. Multivariable logistic regression was used to assess the association between family planning use and food security status after adjusting for other covariates.

**Results:**

Use of modern contraceptive method was significantly low among food insecure women (29.7 %) compared to those who were food secure (52.0 %), (*P* < 0.001). Women from food secure households were nearly twice likely to use modern contraceptive methods (AOR: 1.69 (CI: 1.03, 2.66)). Similarly, those who had antenatal care (ANC) visit (AOR: 4.56 (CI: 2.45, 7.05)); exposure to media (AOR: 4.92 (CI: 1.84, 13.79)) and those who discussed about contraceptive methods with their partner (AOR: 3.07 (CI: 1.86, 5.22)) were more likely to use modern contraceptive methods. Conversely, women who delivered their last child at home were less likely to use modern contraceptive methods (AOR: 0.08 (CI: 0.03, 0.13)).

**Conclusion:**

Food insecurity is negatively associated with modern contraceptive method use. Thus food insecurity should be considered as one of the barriers in designing family planning services and needs special arrangement.

## Background

At the beginning of twenty-first century, world population was estimated to be almost 6.1 billion. According to the United Nation (UN) projection, the world’s population will reach 11 billion by 2020. This continued world population growth has become an urgent global problem. This growth rate is mostly occurring in developing countries where fertility rate is very high [1].

Despite impressive reductions in child mortality and improvements in life expectancy, women’s reproductive health in developing countries particularly Sub-Saharan Africa lags behind leaving birth rates to remain high. Women in the region have on average 5.1 children [2]. Each year, there are an estimated 80 million unintended pregnancies, and 42 million of these pregnancies end in abortion. The primary reason for abortion is to end an unplanned pregnancy. To reduce the number of unintended pregnancies and the number of abortions, women must have access to contraceptive information and services. Studies around the world revealed that, in regions where women had given high-quality contraceptive services, the number of abortions would have been decreased [3].

Family planning is a viable solution to control such a fast growing population and its associated consequences. In addition to spacing and limiting the number of children, it improves the maternal and child health: it empowers women and enhances economic development [4]. Family planning is also one of the human rights. In this regard Article 16 of the Teheran Proclamation issued by the United Nations Conference on Human Rights in 1968 states that "…*Parents have a basic human right to determine freely and responsibly the number and spacing of their children*"[5].

In spite of the massive spending and extensive family planning promotion over three decades, many poor people in the third world remain reluctant to use modern contraception in the early twenty-first century. Attitudes and the need for children among the poor are often quite different from that of family-planning enthusiasts, who are mostly middle class individuals. Mostly when poor people use modern contraceptive methods, their continuation rate of modern contraceptive methods use is often low. Poverty and adverse social conditions including lack of information and access to other methods of birth control, threats of discontinued social benefits, and economic constraints also set the conditions for abuses in family-planning programs [6].

Ethiopia has been deemed a population-climate “hotspot” place where rapid growth and a changing climate pose grave threats to food security and human well-being. One in ten Ethiopians is chronically food insecure, and nearly one in five go hungry in drought years. With almost half of its people under the age of 15 and an average fertility rate of nearly five (4.8) children per woman, Ethiopia’s population is the fifth fastest growing in the world [7]. In Ethiopia most of the modern contraceptive methods that are the results of current technologies are available, and around 97.1 % of the communities are aware of those methods. Modern contraceptive methods that are commonly used in the country are injectables (20.8 %), implants (3.4 %), pill (2.1 %), female sterilization (0.5 %), intrauterine device (IUD) (0.3 %), male condom (0.2 %) and other methods are rarely used [9].

A closer look at Ethiopia shows that neither the Malthusians nor the Boserupians quite get it right concerning the relation between population and food supply. The connections between population and food security are extraordinarily complex. The country is characterized by highly troubling Global Hunger Index (29.8 i.e. 80th out of 84 countries) [8] and poor family planning utilization (only 29 % of married women) [9] which leads to rapid population growth that does not match with available resource [10]. Numbers matter, but so do other dynamics, such as migration and age structure. Context is paramount: the right policies are essential to encouraging and reaping the benefits from positive demographic trends, but those policies must be tailored to local circumstances [7].

Women’s health is crucial to food security and nutrition. Agriculture and food security programmes should be uniquely positioned to respond to women’s productive and reproductive needs. Reproductive health services can improve women’s and children’s nutrition, and better nutrition can improve reproductive health. Population growth directly challenges food security, particularly in sub-Saharan Africa, and food security and agricultural programmes can be an effective means to deliver reproductive health services, and integrate family planning with sustainable agriculture [11].

Addressing the connection between food security and reproductive health is critical to ensure population growth that does not overwhelm the world’s resources. Program for Appropriate Technology in Health (PATH’s) Integrated Population and Costal Resource Management Project exemplifies a cost-effective intervention which integrated sustainable fishing practices with improved access to family planning, enabled coastal communities with a history of rapid population growth, extensive malnutrition, and overwhelmed municipal fisheries to take control of their reproductive health and natural resources for the sustainability of community life [12].

Nearly everywhere, wealthier women are more likely to use modern contraceptives than poorer women. The disparities in use between rich and poor are most pronounced in countries with low contraceptive use such as in Uganda. In countries such as Honduras, contraceptive use overall may rise, but the poor still lag behind [13].

The influence of food insecurity on the utilization of family planning services is not well-explained directly, with little studies demonstrating the effect of socioeconomic status [14], standard of living [15], asset index [16–18] and income [16, 19] on family planning outcome.

There are also incompatible findings from some studies. A study conducted in Butajira District, South Central Ethiopia identified variables like ecology/residence, household food shortage, and educational status and house hold livelihood as determinants of family planning utilization. The study found that married women who were members of food self deficient households were about 1.58 times more likely to use family planning compared to their counterparts in food self sufficient households though the association turned statistically insignificant when other variables are included [20]. Contrary to expectations, spousal communication about family planning and geographic accessibility to service facilities were found to be less critical in determining contraceptive use [16].

Although an overwhelming amount of researches have been given to food security issues and to reproductive health, scanty attention has been given to the relationship between the two. Indeed, there has been some attempt to illuminate how women’s reproductive health status influences household food security, but no enough work has been done in the reverse direction to explicate the relationship between the various aspects of food security and childbearing and family planning utilization. Similarly, there were no studies on the relationship between household food insecurity and family planning utilization particularly in the study area. Thus, this study is designed to comprehend the relationships between food security and reproductive health needs.

## Methods

### Study Area and Setting

A comparative community based cross sectional study was conducted from March 15–30, 2014 in Soddo Zuria Woreda, Wolaita Zone Southern Ethiopia 380 km south of Addis Ababa (The capital of Ethiopia). The study comprised 15 randomly selected Kebeles from 34 rural kebeles (smallest administrative unit in Ethiopia) as there were no urban kebele in the study setting. This Zone is characterized by small landholdings supporting high populations’ and high fertility rates [21].

### Sampling

A sample of 651 married women of reproductive age was included in the study. The sample size was determined using a formula for estimation of two population proportion using Epinfo software with the assumptions of 95 % confidence level, expected proportion of current use of family planning among food secure married women (50 %), power (80 %), the ratio of food secure to food insecure(*r* = 1), Odds ratio (OR) = 2, a design effect of 2 for cluster sampling and a non-response rate of 10 %.

The study subjects were randomly selected from fifteen randomly selected kebeles out of the 34 kebeles. The probability proportional to the size allocation method was employed to determine the number of households to be included from each of the kebeles. The selection of each sampling unit was done by applying systematic sampling method based on the list of households with currently married women available in the database of kebele administration. The data base contains list of households (locally called “Aba Wora”) in the kebele administration that is regularly updated by the administrative bodies through health extension workers of the kebele. The initial household was randomly selected by lottery method using number between one and the sampling interval for each kebeles. Since it was only one woman that was needed per household, in case where there was more than one woman in a given household, a lottery method was employed to identify the women to be interviewed.

### Method of data collection

The questionnaire for determinants of modern contraceptive method was adapted from Ethiopia Demographic Health Survey (EDHS, 2011) English version and further developed by using peer reviewed published literatures to include other determinants of family planning method. Questionnaires constituted information on respondent’s socio demographic and economic variables, food security status, reproductive health, and contraceptive information.

To assure the quality of data, the final English version of the questionnaire was translated by two language experts into the local language of the respondents (Wolaitegna language) and back translated to English in order to keep its consistency. Pre-test was conducted on 5 % of the sample to determine the applicability of the question to be asked on adjacent woreda i.e. Damot Gale Woreda Delbo Kebele before the actual data collection. Correction and modification were done on the instrument after pretest.

Two days training on the objective of the study, data collection tools and interview techniques were given for fifteen Clinical Nurses as data collectors and five BSc nurses as supervisors recruited from health centers. The interview was conducted in a place where the woman felt free to express her feelings and ideas. Moreover, in occasions where the sampled women were not accessed for absence, up to three attempts were endeavored for interviewing to lessen the non response rate. The questionnaires were checked by the supervisors on daily basis for completeness.

### Measurement

Knowledge of modern contraception was measured for at least one modern contraceptive method i.e. if three of the knowledge assessing questions were answered correctly (whether the mother had heard of any modern contraceptive method, knows the importance of modern contraceptive method and knows where to get it). Similarly attitude toward modern contraceptive method was measured using six attitude assessment questions on a five point Likert scale with score values ranging from 1 (strongly disagree) to 5 (strongly agree) adapted from EDHS and other peer reviewed literatures. Respondents that have attitude score greater than the average score for six attitude assessing score using Likert scale were considered as having favorable attitude. Exposure to mass media was measured based on the response of respondents for exposure to at least one media in the last six months. Discontinuation of modern contraceptive method was a unit variable measured by asking about interruption of any modern contraceptive method (MCM) for at least three months after initiation.

The tool for assessment of food security status was adapted from Food and Nutrition Technical Assistance (FANTA) household food insecurity access scale (HFIAS) developed for use in developing country settings, and it is a tool that asks respondents about three domains of food insecurity: (1) experiencing anxiety and uncertainty about the household food supply; (2) altering quality of the diet; (3) reducing quantity of food consumed [22]. The tool consists of nine questions that ask about changes households made in their diet or food consumption patterns due to limited resources to acquire food in the preceding 30 days.

Based on the responses given to the nine questions and frequency of occurrence over the past 30 days, households were given a score that ranges from 0 to 27. A higher HFIAS score is indicative of poorer access to food and greater household food insecurity. For this analysis, households were classified into two groups based on overall distribution of the HFIAS in the sample. The lower the score, the most food secured a household was. Based on the answer to nine occurrence questions and 27 frequency questions; women who responded no to all occurrence questions and those who responded ‘yes’ to the first occurrence question i.e. “In the past four weeks, did you worry that your household would not have enough food?” and responded only ‘rarely’ to frequency questions were classified as food secured.

### Data analysis

The data template format was prepared in Epidata version 3.1 and the data was entered into the software with caution. The completeness of the data were checked. Errors related to inconsistency were verified using data cleansing method. The data were exported to statistical package for social sciences (SPSS) version 20(Illinois Chicago), categorized and sorted to facilitate its analysis.

Descriptive statistics were computed for household food insecurity, socio-demographic characteristics and family planning use. Food-insecure and food-secure households were compared with the logistic regression and chi-square test for proportions through different characteristics of respondents. Some of reproductive health related characteristics of respondents; namely Antenatal care (ANC) follow up, place of delivery of the last child and previous history of child death, were also compared between the two groups. Logistic regression, specifically binary was used to identify factors that were associated with family planning utilization to select variables for multiple logistic regressions. Variables with *p*-value of < 0.25 on binary logistic regression were taken into multivariable logistic regression models to assess the association between independent variables and the outcome variable (modern family planning practice).

Crude and adjusted odds ratios with their corresponding 95 % confidence intervals were computed. A *P*-value ≤ 0.05 was considered statistically significant in this study. Efforts were made to assess whether the necessary assumptions for the application of multiple logistic regression are fulfilled. In this regard, the Hosmer and Lemeshow's goodness-of-fit test was done to check the fitness of the model. Interaction between different predictor variables was checked.

### Ethical considerations

Ethical clearance was obtained from the Ethical Review Committee of Jimma University College of Public Health and Medical Science and letter of permission was obtained from Wolaita Zone Health Office and Soddo Zuria Woreda health office. Informed oral consent was also obtained from each study subject prior to interview and the purpose of the study explained to the respondents.

## Results

A total 651 women were involved in the study giving a response rate of 100 %. Concerning socio-demographic characteristics of the respondents a little over half of the respondents had no education 339(52.1 %).Occupationally more than half of the study subjects 386(59.3 %) were housewives and farmers’ women accounts for 161(24.7 %) of the study subjects. The prevalence of household food insecurity was 60.5 % (Table 1).

**Table 1:** Socio-demographic characteristics of respondents by Food Security Status category; Soddo Zuria Woreda, South Ethiopia, March 2014

Characteristics	Food secure *n*(%)	Food insecure *n*(%)	Total N (%)
Age group (*n* =651)	257	394	651
≤20	12(4.7)	29(7.4)	41(6.3)
21 – 25	14(5.4)	81(20.6)	95(14.6)
26 – 30	81(31.5)	129(32.7)	210(32.3)
31 – 35	61(23.7)	70(17.8)	131(20.1)
35 – 40	63(24.5)	55(14.0)	118(18.1)
≥40	26(10.1)	30(7.6)	56(8.6)
Educational status (*n* =651)	257	394	251
no education	127(49.4)	212 (53.8)	339(52.1)
Primary	112(43.6)	158(40.1)	270(41.5)
Secondary and above	18(7.1)	24(6.1)	42(6.4)
Occupational status (*n* =651)	257	394	651
House wife	158(61.9)	228(57.9)	386(59.3)
Farmer	68(26.5)	93(23.6)	161(24.7)
Merchant	10(3.9)	35(8.9)	45(6.9)
Employed	10(3.9)	16 (4.0)	26(4.0)
Others^a^	11(4.1)	22 (5.6)	33(5.1)
Religion (*n* =651)	257	394	651
Protestant	168(65.4)	229(58.1)	397(61.0)
Orthodox	74(28.8)	116(29.4)	190(29.2)
Catholic	12 (4.7)	40(10.2)	52(8.0)
Others^b^	3(1.2)	9(2.3)	12(1.8)

^a^Daily laborers and others ^b^Muslims and others

Regarding their reproductive health experiences, 15.6 % of food secure mothers and 18.7 % of food insecure mothers reported to have at least one experience of unintended pregnancy and this is relatively lower when compared with national level which is 25 %. Regarding ANC follow up 72.4 % of food secure mothers and 56.7 % of food insecure have ANC visit for their last pregnancy. Nearly three fourth, 73.9 % of mothers reported to have delivered their last child at home, which accounts 65.9 % of food secure mothers and 79.2 % of food insecure. The history of child death was also asked and the respondents reported that 12.4 % of food secure and 16.8 % of food insecure have at least one experience of child death (Table 2).

**Table 2:** Reproductive characteristics of respondents by Food Security Status category; Soddo Zuria Woreda, South Ethiopia, March 2014

Characteristics	Food secured *n*(%)	Food insecured *n*(%)	Total *n* (%)
Age at marriage (*n* = 645)	256	389	645
<18	26(10.2)	74(19.0)	100(15.5)
≥18	230(89.8)	315(81.0)	545(84.5)
230(89.8)			
Mean	20.88 ± 3.3	19.73 ± 2.9	20.19 ± 3.1
Age at delivery (*n* = 604)	244	360	604
<18	7(2.9)	25(6.9)	32(5.3)
≥18	237(97.1)	335(93.1)	257(94.7)
Mean	22.46 ± 3.3	21.23 ± 2.9	21.73 ± 3.1
Unintended Pregnancy (*n* = 624)	250	374	624
Yes	39(15.6)	70(18.7)	109(17.5)
No	211(84.4)	304(81.8)	515(82.5)
ANC follow up (*n* = 624)	250	374	624
Yes	181(72.4)	212(56.7)	393(63.0)
No	69(27.6)	162(43.3)	231(37.0)
Place of delivery (*n* = 620)	250	370	620
Home	164(65.6)	294(79.5)	458(73.9)
Institutional	86(34.4)	76(20.5)	162(26.1)
Child death (*n* = 626)	250	376	626
Yes	31(12.4)	63(16.8)	94(15.0)
No	219(87.6)	313(83.2)	532(85.0)

On behalf of contraceptive characteristics about 38.6 % of women from the study subjects were currently using modern contraceptive method in the study area and it is 52.0 % among women from food secure households and 29.7 % among women from food insecure households. The most common reason for mothers not using modern family planning is a need for more children 28.5 % followed by religious prohibition 20.3 %. About 96.9 % of women from food secure households and 86.8 % of food insecure had knowledge of modern contraceptive method. Information about exposure to mass media shows that 95.6 % of women from food secure households and 82.5 % of women from food insecure households had history of exposure to at least one media within the last six months (Table 3).

**Table 3:** Modern contraceptive method related information of respondents by Food Security Status of the respondents; Soddo Zuria Woreda, South Ethiopia, March 2014

Characteristics	Food secure *n*(%)	Food insecure *n*(%)	Total *n* (%)
Media Exposure (*n* = 633)	250	383	633
Yes	239(95.6)	316(82.5)	555(87.7)
No	11(4.4)	67(17.5)	78(12.3)
Attitude toward MCM(*n* = 633)	250	383	633
Favorable	242(96.8)	362(94.5)	604(95.4)
Unfavorable	8(3.2)	21(5.5)	29(4.6)
MCM use (*n* = 632)	252	380	632
Yes	131(52.0)	113(29.7)	244(38.6)
No	121(48.0)	267(70.3)	388(61.4)
Ever use of MCM (*n* = 629)	250	379	629
Yes	203(81.2)	283(74.7)	486/(77.3)
No	47(18.8)	96(25.3)	143(22.7)
Discontinuation of MCM (*n* = 486)	203	283	486
Yes	75(36.9)	143(50.5)	218(44.9)
No	128(63.1)	140(49.5)	268(55.1)
Knowledge MCM (*n* = 651)	257	394	651
Yes	249(96.9)	342(86.8)	591(90.8)
No	8(3.1)	52(13.2)	60(9.2)

On bivariate analysis some of the respondent’s characteristics show variation between food secure and food insecure households. The proportion of women marrying before 18 years of age is higher for food insecure households, *p* = 0.002. On the other hand the proportion of women having unintended pregnancy was insignificantly different between the two groups: 15.6 % among food secure and 18.7 % among food insecure, (*P* = 0.16). Majority of women from food secure households (72.4 %) had at least one ANC visit compared to women from food insecure households which was 56.7 % (*P* < 0.001). The proportion of women who have delivered their last child at a health institution was significantly smaller among food insecure households (20.5 %) as compared to women from food secure households (34.4 %) with (*P* < 0.001). The proportion of women using modern contraceptive method was significantly different among women from food secure (52.0 %) and food insecure (29.7 %) households (*p* < 0.001) and among ever users of modern contraceptive method discontinuation rate is higher for women from food insecure households (50.5 %) compared to food secure households (36.9 %) with (*P* = 0.003) (Table 4).

**Table 4:** Respondents reproductive and contraceptive related characteristic by food security status Zuria Woreda, South Ethiopia, March 2014

Variables	Food secure *n* (%)	Food insecure *n* (%)	X^2^	P
Age at marriage(*n* = 645)	256	389		
<18	26(10.2)	74(19.0)	9.2	0.002
≥18	230(89.8)	315(81.0)		
Unintended Pregnancy(*n* = 624 )	250	374		
Yes	39(15.6)	70(18.7)	1.956	0.162
No	211(84.4)	304(81.8)		
ANC^a^ follow up (*n* = 624 )	250	374		
Yes	181(72.4)	212(56.7)	12.43	≤0.00001
No	69(27.6)	162(43.3)		
Place of delivery(*n* = 620 )	250	370		
Home	164(65.6)	294(79.5)	14.85	≤0.00001
Institutional	86(34.4)	76(20.5)		
History of Child death(*n* = 626 )	250	376		
Yes	31(12.4)	63(16.8)	2.23	0.14
No	219(87.6)	313(83.2)		
MCM^b^ use(*n* = 632 )	252	380		
Yes	131(52.0)	113(29.7)	31.64	≤0.0001
No	121(48.0)	267(70.3)		
Discontinuation of MCM (*n* = 486)	203	283		
Yes	75(36.9)	143(50.5)	8.82	0.003
No	128(63.1)	140(49.5)		
Discussion MCM(*n* = 643 )	256	387		
Yes	167(65.2)	200(51.7)	11.555	0.001
No	89(34.8)	187(48.3)		
Knowledge MCM(*n* = 651 )	257	394		
Yes	249(96.9)	342(86.8)	18.90	≤0.0001
No	8(3.1)	52(13.2)		

^a^ANC = Antenatal care ^b^MCM = Modern contraceptive method

Concerning the association between modern contraceptive utilization and respondents characteristics, the results from binary logistic regression showed that household food security (*P* < 0.001), ANC visit (*P* < 0.001), place of delivery (*P* < 0.001), knowledge of modern contraceptive method (P < 0.001), attitude toward modern contraceptive method (*P*- < 0.001), exposure to media (*P* < 0.001), discussion with partner (*P* < 0.001) and total number of birth (*P* < 0.001) were significantly associated with modern contraceptive method (Table 5).

**Table 5:** Bivariate model of factors associated with modern contraceptive utilization of respondents by category; Soddo Zuria Woreda, South Ethiopia, March 2014

Characteristics	Current users	Non-users	COR [95 % CI]
	*n*(%)	*n*(%)	
Household food security (*n* = 632)	244	388	
Food secure	131(53.7)	121(31.2)	2.56 (1.84, 3.56)
Food insecure	113(46.3)	267(68.8)	1.00
ANC follow up(*n* = 608)	242	366	
Yes	199(82.2)	186(50.8)	4.48(3.04, 6.60)
No	43(17.8)	180(49.2)	1.00
Place of delivery(*n* = 604)	241	363	
Home	115(47.7)	332(91.5)	0.085(0.05, 0.13)
Institutional	126(52.3)	31(8.5)	1.00
Knowledge of MCM(*n* = 632)	244	388	
Yes	231(94.7)	349(89.9)	2.10(1.01, 4.36)
No	13(5.3)	39(10.1)	1.00
Exposure to mass media(*n* = 628)	250	386	
Yes	229(95.4)	326(84.0)	3.959(2.04, 7.68)
No	11(4.6)	62 (16.0)	1.00
Attitude of MCM(*n* = 628)	240	388	
Favorable	238(99.2)	361(93.0)	8.9(2.10, 37.78)
Unfavorable	2(0.8)	27(7.0)	1.00
History of Child death (*n* = 610)	241	369	
Yes	29(12.0)	59(16.0)	0.72(0.45, 1.16)
No	212(88.0)	310(84.0)	1.00
Discussion with their husband on MCM(*n* = 631)	243	388	
Yes	181(74.5)	174(44.8)	3.59(2.53, 5.10)
No	62(25.5)	214(55.2)	1.00
Total Number of birth(*n* = 606)	241	365	
3 and below	114(47.3)	227(62.2)	1.00
3 to five	87(36.1)	90(24.7)	1.659(1.031, 2.671)
above five	40(16.6)	48(13.2)	0.862(0.516, 1.44)

After adjusting all the other variables in the multivariable logistic regression model household food insecurity, delivery place of the last child, religion of the respondent, media exposure, antenatal care (ANC) follow up and discussion with husband about modern contraceptive methods (MCM) of the respondents were independently associated with modern contraceptive use. Women from food secure households were 1.7 times more likely to use MCM compared to women from food insecure households [adjusted odds ratio (AOR): 1.69, (confidence interval (CI): 1.03, 2.66). Mothers who had at least one ANC follow up were nearly five times more likely to use MCM when compared to women who have no any ANC visit [AOR: 4.56, (CI: 2.45, 7.05)]. Concerning place of delivery, the likelihood of using MCM decrease by 92 % for those women who delivered their last child at home compared to institutional delivery, [AOR: 0.08 (CI: 0.03, 0.13)]. Exposure to mass media has also significant effect on MCM utilization. Women who were exposed to at least one media in the last six months were about 5 times more likely to use MCM than those who have no any history of exposure [AOR: 4.92, (CI: 1.84, 13.79)]. Discussion between couples is also positively associated with modern contraceptive method utilization [AOR: 3.07, (CI: 1.86, 5.22)] (Table 6)

**Table 6:** Predictors^a^ of modern contraceptive use among women in reproductive age group Soddo Zuria Woreda, South Ethiopia, March 2014

Variables	Current use of modern contraceptive	Current use of modern contraceptive	Adjusted OR(CI)
	Yes	No	
	no. (%)	no. (%)	
Food security status (*n* = 632 )	244	388	
Food secured	131(53.7)	121(31.2)	1.69(1.03, 2.66)
Food insecured	113(46.3)	267(68.8)	1.00
Discussion on MCM (*n* = 631 )	243	388	
Yes	181(74.5)	174(44.8)	3.07(1.80, 5.20)
No	62(25.5)	214(55.2)	1.00
ANC follow up (*n* = 608 )	242	366	
Yes	199(82.2)	186(50.8)	4.56(2.64, 7.05)
No	43(17.8)	180(49.2)	1.00
Exposure to mass media (*n* = 628)	240	388	
Yes	229(95.4	326(84.0)	4.92 (1.84, 13.79)
No	11(4.6)	62 (16.0)	1.00
Place of delivery(*n* = 604)	241	363	
Home	115(47.7)	332(91.5)	0.08 (0.03, 0.13)
Institutional	126(52.3)	31(8.5)	1.00

^a^Controlled for age, educational status, occupation, religion, age at marriage, age at delivery, number of currently alive children, knowledge, and source of income

## Discussion

In Ethiopia only three in every ten (29 %) of currently married women were using contraceptive method. About 93 % of these methods were modern methods i.e. 27 % out of 29 %. About twenty-five percent of currently married women have an unmet need for family planning services [9]. In this study the prevalence of modern contraceptive method use is relatively higher than the national and regional contraceptive prevalence level. It is also relatively higher than the prevalence in rural area of Butajira from previous study done on determinants of low family planning use and high unmet need [20]. Similarly, from our study other maternal health services like ANC and institutional delivery are also higher than the national level.

The result of this study showed that women from food secure households were about 1.7 times more likely to use modern contraception than women from food insecure households. Although there were no studies that show the relation between food insecurity and modern contraceptive use some previous studies show that poorer women were less likely to use modern contraceptive method and women with higher income are more likely to use contraception compared to the poor women [16]. In study done on utilization of family planning services by married Sudanese women of reproductive age; women with a higher socioeconomic status were found to be more likely to use modern methods of family planning than their counterparts [14]. In addition to this when poor people use modern contraceptives, their continuation rates are also often low [6]. Among ever users of modern contraceptive in this study larger number of women from food insecure households, about 50.5 %, had discontinued using modern contraceptive method as compared to women from food secure households which is only 36.9 % had discontinued using modern contraceptive method.

This finding has important implication especially in Ethiopia, which is one of the world’s most food insecure countries with problems along all key dimensions of food security[22] where one in ten Ethiopians is chronically food insecure. With almost half its people under the age of 15 and an average fertility rate of nearly five (4.8) children per woman, Ethiopia’s population is the fifth fastest growing in the world [7]. The combined effect of high prevalence of food insecurity and a youthful population with potential for population momentum poses arguments on the need for devising appropriate intervention strategies to promote use of modern family planning methods especially in food insecure areas to curb the synergistic negative consequences of both problems.

The study also found that women who have discussion with their husband were about 3 times more likely to use modern contraceptive method. Spousal discussion about family planning and contraceptive practice was documented to be crucial for the wider acceptance of contraceptive practice and lessening partners’ fertility intention in developing countries [23, 24]. A study done on awareness and determinants of family planning practice in Jimma, Ethiopia indicated that the percentage of women who used modern contraceptives was higher among those who had discussed family planning with their husbands than among those who had not [25]; in agreement with the study done in Butajira that showed a positive association between couple’s discussion on family planning and contraception [20].

Utilization of other health services like previous attendance of ANC, post natal care (PNC), and delivery at health institution and attendance of immunization services were found to have statistically significant association to the current use of modern contraception. In this study ANC follow up and institutional delivery were found to be statistically significant. Regarding ANC follow up and modern contraceptive method use the women who have at least one ANC follow up are more likely to use modern contraceptive method compared to those who have no any ANC follow and women who delivered at home were also less likely to use modern contraceptive compared to those that delivered at health institution. It may be that women who deliver at health institutions receive more exposure to family planning information compared to those who deliver at home. Information about public exposure to messages through a particular medium allows policy makers to ensure the use of the most effective means of communication for various target groups in the population and it is one of the enabling factors for proper utilization of modern contraceptive method [9]. In this study women that have exposure to media were about five times more likely to use modern contraceptive method. In study done in Wolaita Soddo Town south west Ethiopia women who have radio and television were about three times more likely to use contraceptive method as compared to those who have not [26].

The study on family planning knowledge and current use of contraception among the Mru indigenous women in Bangladesh also shows that women who have exposure to mass media were six times more likely to use contraceptive methods [27]. Study done on factors influencing contraceptive use among young women in urban squatter settlements of Karachi, Pakistan stated that women were more likely to use contraceptives when messages of family planning were delivered through media [28].

In Ethiopia national health policy gives a high priority to the democratization and decentralization of the health service systems and one of targets of the Ministry of Health, with respect to improving maternal and child health, is to increase the contraceptive prevalence rate (CPR) to 66 percent by 2015 [29]. This study showed that households’ food insecurity significantly affects modern contraceptive method use and its continuation. Hence any program whether governmental or non-governmental that wants to improve modern contraceptive method use and its continuation should consider food security status of the area while planning for family planning service and should cooperatively work with agricultural offices.

Strong emphasis should be given to active involvement of women in productive activities like Productive Safety Net Program that is being practiced in the country with the aim of enabling the rural poor facing chronic food insecurity to resist shocks, create assets and become food self sufficient. Woreda Health Office should also give emphasis to modern contraceptive use related reproductive health services like ANC and place of delivery. ANC follow up and institutional deliveries show significant improvement in modern contraceptive method use. Therefore improving the rate of ANC follow up and institutional delivery may highly improve utilization of modern contraceptive method. Awareness creation on the importance of discussing on reproductive health issue for both mothers and their husbands and encouraging women in discussions about modern contraceptive methods with health professional should be encouraged.

### Strength and limitation

There were no non respondent from the sample and all of the probably selected individuals were interviewed. The study was comparative in nature and efforts were made to show the difference in different characteristics of the respondents in the two groups. By controlling for confounders as much as possible the difference in MCM utilization among the two groups was assessed. We admit a number of limitations in our study. Although we used standardized tool to assess food security status there may be possibility of some misclassification. Household Food Insecurity Access Scale measures acute food insecurity as it asks food access within the last one month. In addition, our analysis was based on cross-sectional data whereas data capturing seasonal trends are needed to fully understand the relationship between modern contraceptive use and household food insecurity. The cross-sectional nature of the data also limits our ability to draw any causal conclusions i.e. failure to use modern contraceptive that leads to large family size resulting in food insecurity may be the case.

## Conclusion

Overall, the study showed that food insecurity is a significant barrier to use of modern contraceptive methods. Not only family planning method use, but also women from food insecure households were less likely to practice other reproductive services like antenatal care and institutional delivery. Food security interventions should integrate appropriate strategies for enhancing use of modern contraceptive methods in food insecured areas.

## Abbreviations

EC: Ethiopian calendar
FAO: Food and Agricultural Organization
FP: Family planning
HIV/AIDS: Human immunodeficiency virus/ Acquired immunodeficiency syndrome
IUDs: Intrauterine devices
NGO: Non-Governmental Organization
MFP: Modern family planning
PNC: Post-natal care
SNNPR: Southern Nations Nationalities and Peoples Representative
UN: United Nations
WHO: World Health Organization
PATH: Program for appropriate technology in health
